# Diversification of Morphological Features of the Dark European Honey Bee of the ‘Augustow M’ Line

**DOI:** 10.3390/ani11041156

**Published:** 2021-04-17

**Authors:** Beata Madras-Majewska, Lucja Skonieczna

**Affiliations:** 1 Apiculture Division, Institute of Animal Science, Warsaw University of Life Sciences, Nowoursynowska 166, 02-787 Warsaw, Poland; 2 Owner of the Breeding Apiary Sawa, Helenowska 22/28, 05-804 Pruszkow, Poland; I.skonieczna@interia.pl

**Keywords:** native middle-European bee, morphological features, feature diversification

## Abstract

**Simple Summary:**

The necessity of protecting the Dark European honey bee (*Apis m. mellifera*) in Poland was realized in the 1970s. This was a consequence of the displacement of native bees caused mainly by the mass importation of queen bees of foreign species. Today, we have four programs for the conservation of the following lines of our Dark European honey bee: Augustow M, Kampinos M, Asta M and Northern M. These programs aim to keep the bee gene pool as rich as possible and to preserve the phenotypic and behavioral features characteristic of the *Apis m. mellifera* species. The aim of this study was to investigate the diversification of morphological features of the Dark European honey bee of the Augustow M line. The authors have demonstrated that the morphological features of the Augustow M line—crucial for identifying the species affiliation, determined as part of the authors’ research—are consistent with the corresponding features described by relevant Polish references used for the conservation of native bee lines.

**Abstract:**

The aim of this study was to investigate the diversification of morphological features of the Dark European honey bee of the Augustow M line. The authors studied the proboscis length and cubital index, as features determining the affiliation to the species; the width of tergite 4 and the sum of widths of tergites 3 + 4, as indicators of the bee body size; and the length and width of the right forewing. They compared bees sampled from (1) the “lead apiary”, (2) “associate apiaries” and (3) “conservation area apiaries”—apiaries situated in the conservation area established by the national program for the conservation of genetic resources of this bee line. The conclusion was that it is possible to protect bees of the Augustow M line under the existing program, based on resources available to the lead, associate and conservation area apiaries. The bees studied have the essential features of the Dark European honey bee and the values of parameters tested are consistent with the morphological feature references valid for *Apis m. mellifera*. On the other hand, based on the authors’ research and on other studies described in literature of 1960s, there is a dwarfing trend in the Dark European honey bee of the Augustow M line.

## 1. Introduction

The work to describe European bee populations and their diversity more thoroughly was inspired by the research of Alpatov [1,2,3,4] and Goetze [5]. Morphometric studies of phenotypes and of the diversification of these bees made it possible to describe changes in the bee body dimensions depending on latitude [4,6]. Sophisticated statistical analyses of these morphological features carried out by further researchers in 1975 to 1986 made it possible to distinguish the *Apis mellifera* subspecies and to locate their habitats [7,8,9,10].

Most of the territory of Poland was inhabited by bees showing features typical for *Apis m. mellifera*, while a population more related to forest bees (*Apis m. silvarum Scor.*) lived in the former Bialystok province [11]. Morphometric studies have identified four distinct populations of Dark European honey bee living in Poland.

The continuing degradation of the environment, parasites, pathogens and genetic isolation, combined with inbreeding, have increased the mortality rates in bees. [12,13]. These factors have indirectly contributed to a sharp drop in honey bee populations, including the middle-European bee. The mass importation of queens of the *Apis m. caucasica* and *Apis m. carnica* species, and the introduction of these species to domestic apiaries for economic reasons, was an additional factor that has drastically increased hybridization and depopulated the hives of *Apis m. mellifera*. Accordingly, the area of Poland inhabited by the Dark European honey bee has shrank over the recent decades to a few spots located in the north-eastern and central parts of the country where the insects are now kept under protection. The distribution and extent of the presence of the Dark European honey bee in Poland have been described in detail by Bornus et al. [11] and by Ruttner [14,15,16].

Today, considering the threat of extinction of *Apis m. mellifera*, conservation of native bees in their original habitats is critical. The efforts to sustain this population aim to preserve the unique characteristics of the Dark European honey bee while preserving as vast a genetic variety as possible. The safeguarding of native *Apis m. mellifera* against hybridization has been considered a general ecological goal that can be achieved through the establishment of preserves friendly to bees in terms of their reproductive biology [17]. On the formal part, the implementation of this protection became possible in 1975, after the establishment of the Central Animal Breeding Station (the National Animal Breeding Center as of 2001) with its local branches as the supervisors of breeding apiaries Troszkiewicz [18] and after recognition of the bee as a “farm animal” (under the regulation of the Council of Ministers of 10 July 1975, Journal of Laws 26, item138).

Conservation programs are underway for four lines of Dark European honey bee: Augustow M, Kampinos M, Northern M and Asta M. For two lines (Augustow M and Kampinos M), their programs rely on dedicated conservational breeding areas and on the collaboration of lead, associate and conservation area apiaries. The two other programs build just on collaboration between lead and associate apiaries. The implementation of these programs is supervised by the Ministry of Agriculture through the State Zootechnical Research Institute, the National Animal Breeding Center and the Horticultural Institute. The latter carries out research projects (numbers 21–24) for biological advancement in animal production [19,20,21,22,23], (http://www.inhort.pl/projety-badawcze/projektyfinansowane-przez-mrirw/badania-podstawowe-na-rzecz-postepu-biologicznego-w-produkcji-zwierzecej, accessed on 10 January 2021). Validity of the assumptions underlying the programs seems to be corroborated by the latest COLOSS studies (2014–2016), according to which local populations of bees, including *Apis m. mellifera*, are adapted to their environments and can handle diseases, parasites and other pathogens and the establishment of conservation spots for threatened populations can be one of the ways of achieving the goal [24,25,26,27].

The Augustow M line is the most ancient and most numerous population among middle-European bee lines protected in Poland. There is a 1100 km^2^ area for the conservational breeding of this line in the Augustow Forest, established by the governor of Suwalki province in 1976, and continuing without interruption until the present (recently upheld by regulation 56/05 of the Podlasie Province governor on 7 July 2005; http://bip-archiwum.bialystok.uw.gov.pl/bip/Information.aspx?iid=4275, accessed on 5 January 2021).

This refuge has the shape of a circle of an approximate 30 km diameter. The circle has a bull’s eye, the Plaska locality, and two rings: the inner of a 5 km radius, and outer of a 10 km radius. The inner ring, effectively isolated by forest complexes and by topography, is reserved for the artificial insemination of queen bees, while natural insemination is also possible. Bees kept in this ring are the source of genes for the lead apiary. The middle ring isolates the inner one from foreign genes. For this purpose, the middle ring is populated with as many non-inseminated queen bees of the Augustow M line, coming from the lead apiary, as possible. By mating within this isolation zone, queens lock foreign genes out from the inner ring and, at the same time, increase the probability of desirable insemination (by producing pure-line drones). The genetic material is exchanged in two ways between the lead apiary, on the one hand, and the associate and conservation area apiaries, on the other hand. The lead apiary sources only such queens that meet the reference requirements for the line and distributes quality genetic material to the associate and conservation area apiaries [20,21].

Bees introduced to the conservation area are strictly selected based on their confirmed affiliation to the Augustow M line to minimize the risk of contamination of the protected populations with foreign genes (e.g., as a result of the intrusion of drones or migration of swarms). The main criteria for the selection include body color, glossa length, cubital index value and tergite 4 width or the sum of widths of tergites 3 + 4 [28].

Various methods, evolving over time, were used to study the morphological features of bees. The standard morphometry was employed by many researchers [11,29,30]. The development in computer-aided techniques made it possible to elaborate the geometric morphometry method that has been readily adopted by many researchers [31,32,33,34,35,36,37,38,39,40,41]. However, comparisons of the two methods have revealed that the geometric morphometry is just slightly more effective than the standard one in the discrimination between bee subspecies [40]. Then, Bustamante et al. [42] found that the application of the geometric morphometry for the comparison of two bee subspecies was less accurate (73.7%), even taking into account data after studying all wings in comparison to the analysis of data obtained using the standard morphometry (97%). DNA tests are often used to eliminate hybridized specimens from protected populations [40,43,44] but a selection based on DNA markers as the single criterion can lead to a loss of genes making the phenotype. This means that the DNA test (identifying diagnostic alleles for the *Apis m. mellifera*) and the phenotypic analysis (recognizing alleles decisive for the characteristic phenotypic features of the Dark European honey bee including the Augustow M line) should go hand in hand.

Even though we have a number of methods, the conventional morphological evaluation has not become any less important. Researchers still value the method and use it willingly [40,42,45]. Note that the standard morphological method makes it possible not only to reliably measure such features as the body size or proboscis length but also to track the diversification of such features. This is very important for programs for the conservation of genetic resources of the Dark European honey bee including the Augustow M line. Additionally, references and models generally adopted and valid for the *Apis m. mellifera*, developed in, and applied since, the 1960s, are still used for the determination of the species affiliation of the Augustow M line bees [18,36,46].

The purpose of the authors’ study was to analyze the diversification of morphological features of the native Dark European honey bee of the Augustow M line. Furthermore, the study aimed to verify whether the program for the conservation of genetic resources of the Augustow M line, based on collaboration of the lead, associate and conservation area apiaries, can sustain the distinct features of the Dark European honey bee and whether the program follows the references valid for *Apis m. mellifera*.

## 2. Materials and Methods

The experimental material consisted of specimens of the Dark European honey bee (*Apis m. mellifera*) of the Augustow M line, covered by the program for the conservation of genetic resources of bees in Poland, sampled over ten successive years. The specimens were sourced from the Breeding Apiary in Parzniew (formerly owned by the Animal Breeding and Insemination Station in Bydgoszcz and currently by the National Board of Agricultural Chambers). The stationary apiaries were operated in an extensive manner. The apiaries performed standard treatments of the hive management method with a relatively poor forage. The health of the honeybee colony was regularly monitored and standard procedures were used to combat varroasis.

Bees were sampled from 265 bee colonies, the following nine lead, associate and conservation area apiaries breeding the Augustow M line as part of the conservation program:

1. Lead apiary (locality: Plaska);
2. Associate apiaries (localities: Jasionowo, Bryzgiel, Lipsk, Danowskie);
3. Conservation area apiaries (localities: Plaska Municipality, Rubcowo, Sucha Rzeczka, Muly).

The multistage clustering method (cluster draw) [47] was used for the sampling in the successive years of random bee specimens from formerly selected random families. A different number of specimens were collected in each year, so the “system” was unbalanced.

### 2.1. Morphological Analyzes

**Figure 1:**
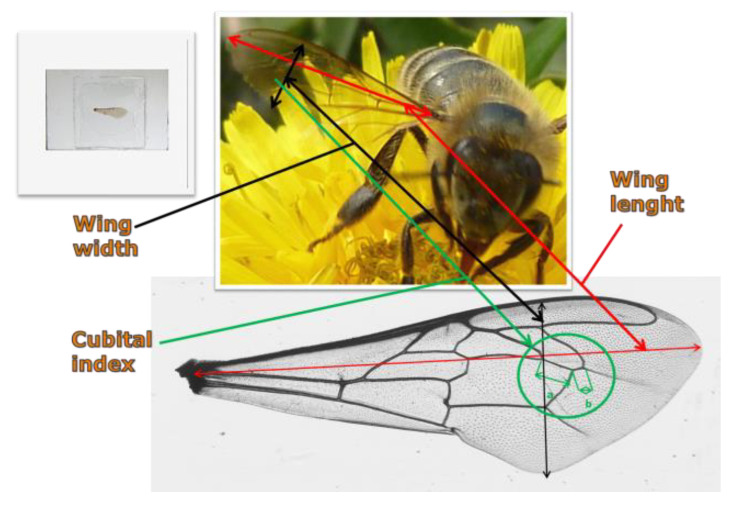
Images 1–3 morphometric measurement site—wing width, wing length, cubital index.

**Figure 2:**
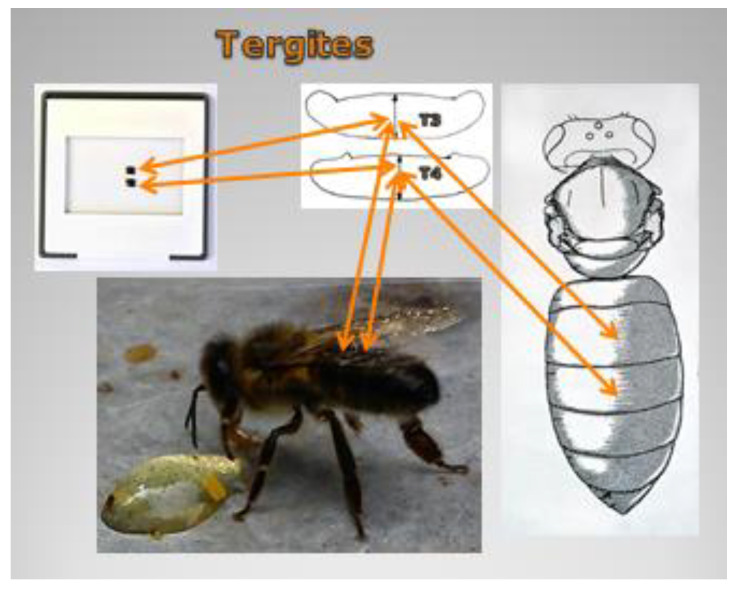
Images 4–7 place of morphometric measurement—tergites.

**Figure 3:**
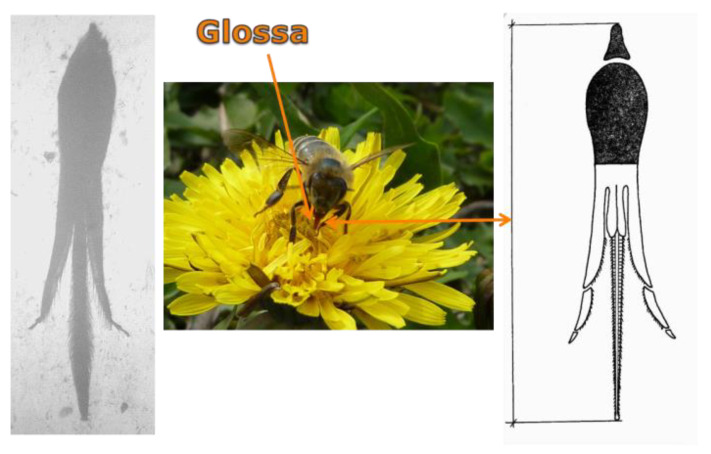
Images 8–10 place of morphometric measurement—glossa.

Specimens placed in the Fotie boxes were thrown into boiling water to straighten up glossa for measurement. Then, the specimens were drained, placed in glass containers in groups of 30 and preserved with 70% ethyl alcohol (supplier: Poch S.A., Warsaw, Poland). The right forewing (further referred to as the “wing”), abdominal tergites 3 + 4 and glossa were collected and prepared for each specimen as an input to seven measurements. Four measurements were taken for the wing (length, width and cubital index) shows images 1–3 (Figure 1), two for the abdomen (widths of tergites 3 + 4) shows images 4–7 (Figure 2) and one for the glossa shows images 8–10 Figure 3). In aggregate, 6927 worker bees were examined by 48,489 measurements.

**Figure 4:**
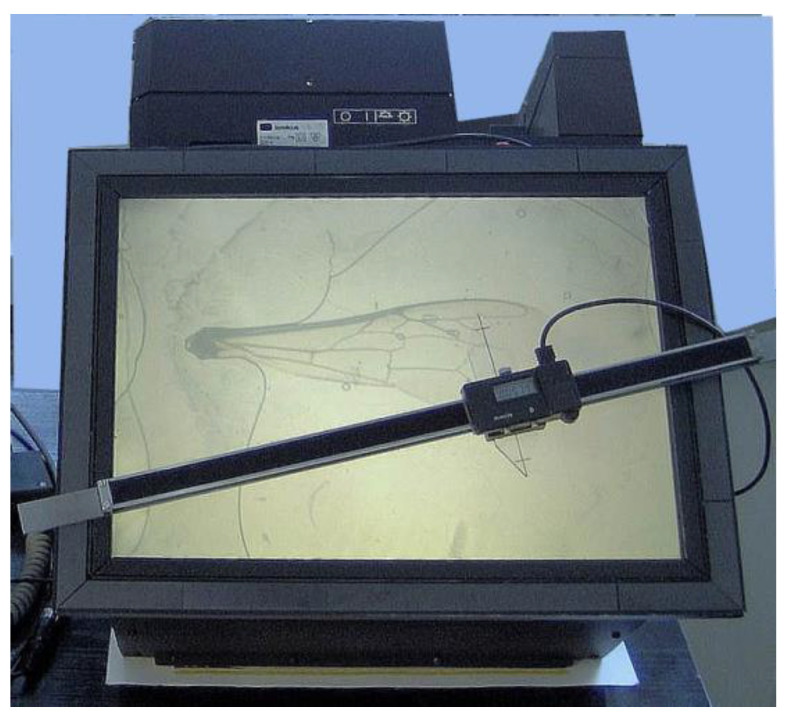
Image 11 apimetr instrument dedicated to morphometric measurements.

Prepared body parts of each specimen were placed on three slide frames and covered with microscopic cover slips glued to the frames: one for the wing, one for the 3 + 4 tergites and one for the glossa. The morphological measurements were performed on the frames loaded in the Apimetr instrument (custom-manufactured by Polskie Zakłady Optyczne/Polish Optical Company/; equipment dedicated to morphometric measurements; device data: electronic slide caliper—measurement accuracy 0.01 mm, slide projector with asymmetric lens eliminating distortion of the image of elements measured on the screen of the apimetr—magnification 20_×_ optical zoom) as shown in image 11 (Figure 4). Images of body parts displayed by this instrument in a 20_×_ optical zoom were measured with a special perspex jaw caliper. The readouts were entered in the computer database as actual dimensions.

### 2.2. Statistical Analyses

A unifactorial analysis by the mean least squares method, carried out by means of the PASW Statistics 23 (2020) software suite, was used for the statistical elaboration of the results. After initial statistical analyses, the description of the study took account of only statistically significant relations between various factors (*p* < 0.01 or *p* < 0.05). Pearson correlation coefficients were computed for the examination of relations between the seven morphological features of the Dark European honey bee of the Augustow M line.

## 3. Results

The wing length, wing form factor and glossa length of the Dark European honey bee of the Augustow M line had the smallest coefficient of variation among the features tested (Table 1). The parameter of the wing length was 56% and 49% smaller than the variation coefficient for the wing width and for the width of tergite 4, respectively. The values of the coefficient of variation were similar for the wing width, for the width of tergite 4 and for the sum of tergite 3 + 4 widths—the variation ranged from 2% to 16%.

The Augustow M line conservation program keeps the breeding material in the lead, associate and conservation area apiaries. This distribution has been addressed in the elaboration of the results (Table 2).

Based on Table 2, bees sampled from the associate apiaries had longer and wider wings than bees from the lead and conservation area apiaries: 0.6% and 0.55% longer and 2.3% and 0.93% wider, respectively. Likewise, bees from the associate apiaries had wider tergite 4 and a larger sum of the widths of tergites 3 + 4. The mean width of tergite 4 was 1.85% and 0.4% larger than those for the lead and conservation area apiaries, respectively. The same was the case for the sum of the widths of tergites 3 + 4 (1.4% and 0.5%) and for the cubital index (1.8% and 4.63%). Just one parameter, the glossa length, broke the pattern: specimens from the conservation area had a length 0.01% and 0.9% longer than those from the associate and lead apiaries, respectively.

Associate apiaries kept the breeding material by following the same methods as the lead apiary, so morphological features were compared for both the apiary types taken together (Table 3).

There were statistically significant differences only between the wings of the specimens. The wings of bees from the associate apiaries were significantly longer (0.029 mm) and wider (0.02 mm) than of those from the lead apiary. There were no statistically significant differences for the remaining features. This legitimized the treating of these two apiary types as a single group and comparing the typical features of their bees to those of bees from the conservation area apiaries (critical for the Augustow M line conservation program) (Table 4).

Bees from the conservation area apiaries had significantly longer wings (0.03 mm) and wider tergite 4 (0.013 mm) than bees from the two other apiary types. The remaining differences turned out to be statistically insignificant (Table 4). Pearson correlation coefficients were computed for the examination of relations between the seven morphological features of the Dark European honey bee of the Augustow M line. The correlations were estimated for the same number of degrees of freedom (df = 5748) (Table 5). Only in two cases did the correlations turn out to be statistically insignificant: those between the wing length and the cubital index and between the bee size (the sum of widths of tergites 3 + 4) and the cubital index. The values of the coefficient *r* for these relations was negative and close to null. The correlation between the wing width and the form factor was weak but statistically significant (r = 0.026 at *p* = 0.0447) as in the case of the negative correlation between the width of tergite 4 and the cubital index (r = −0.030 at *p* = 0.0219). The remaining relations were statistically highly significant. A negative and equally strong correlation existed between the wing form factor and the wing width. It was interesting to discover weak, but statistically highly significant, correlations between the glossa length and the bee size (both the width of tergite 4 and the sum of widths of tergites 3 + 4, for which *r* ranged from 0.191 to 0.202). It is also notable that there are weak but highly significant negative correlations between the wing form factor and the bee size (tergite 4 width) and between the wing form factor and the glossa length, for which the coefficient *r* was equal to −0.266 and −0.251, respectively (Table 5).

## 4. Discussion

The study investigated the morphological diversification of the Dark European honey bee of the Augustow M line covered by the conservation program. The cubital index is one of the features recognized by researchers as enabling the verification of the affiliation of a given bee population to a specific subspecies. The mean values of the cubital index for the Danish population of Dark European honey bees range from 1.580 to 1.880 [16]. The mean value of this parameter determined by the authors for the Polish Augustow M line—first without the split into the apiary types (1.664) (Table 1) and, then, taking into account the split into the lead apiary (1.665), associate apiaries (1.695) and conservation area apiaries (1.620) (Table 2)—fits within this range. The foregoing results are also similar to the figure established by Goetze [6] for bees in Germany (1.690). On the other hand, bees from the Polish conservation area and associate apiaries are 5.2% and 10.1%, respectively, larger than bees in Russia (cubital index 1.540) [2]. At the same time, the foregoing figures coming from the author’s research are 6.4% smaller than the largest mean value of the index for the Augustow M line (associate apiaries) and smaller than the bottom value determined for Lithuanian bees [48] (Table 2). The values of the cubital index for bees in French Brittany [7,49] and in Cavennes [50] ranged from 1.760 to 1.780 and from 1.70 to 1.88, respectively. According to Ruttner [16], the cubital index value was 1.721 for bees in Austria and 1.840 for western-European bees known as “black bees”. By comparing the values of the cubital index for France, Austria and Germany, the authors found that bees from these regions had values 4.9 to 11% larger than bees of the Augustow M line (Table 1 and Table 2).

The mean values of the glossa length for all bees of the Augustow M line are as follows: without the split into the apiary types—6.104 mm; with the split into the lead, associate and conservation area apiaries—6.082 mm, 6.137 mm and 6.138 mm, respectively. The results of the authors’ research are consistent with the values determined by Ruttner [10], 5.8 to 6.4 mm; and by Ruttner et al. [16], 5.950 to 6.190 mm (Table 1 and Table 2).

The mean values of the sum of the widths of abdominal tergites 3 + 4 in studied bees, established by other researchers, are as follows: Alpatov [2]—4.798 mm, Misis [48]—4.660 to 4.840 mm, Ruttner et al. [16]—4.522 to 4.676 mm. These values are consistent with those determined by the authors for the Augustow M line, without the split into the apiary types (Table 1) and with the split (Table 2). Similarly, the authors’ results for the size (the sum of widths of tergites 3 + 4) of bees in Poland coincides with the findings of Gromisz [51], 4.730 to 4.90 mm; and Prabucki and Mickiewicz [52], 4.78 mm. According to earlier studies, Dark European honey bees in Poland used to be larger in the 1960s and 1970s. The mean sum of tergites 3 + 4 ranged from 4.790 mm to 4.990 mm for bees from Szepietow and Konskowola [53] and was equal to 4.850 mm for bees living in northern Poland [54]. Then, in the 1980s, the size of *Apis m. mellifera* in Poland, measured by the width of abdominal tergite 4, decreased to 2.356 mm [55] and, later on, for the Augustow M line, according to the authors’ research, to 2.284 mm (Table 1). This shows a clear dwarfing trend.

The mean values of the cubital index for bees studied between 1971 and 1999 are consistent with the results of the authors’ research but were higher by 0.186 in 2009 (Table 6). Likewise, the comparison of the mean glossa length determined, contemporaneously with the cubital index, by other researches—Bornus [53], 6.238 mm; Gromisz [51], 6.120 to 6.230 mm; Gromisz [55], 6.115 mm; Gromisz and Bornus [54], 6.149 mm; Gromisz and Platek [56], 6.151—to the results of the authors’ research supports the claim that this parameter has not changed since 1960s.

The implementation of the breeding programs in Poland is based, among others, on the application of the mathematical-morphological models for individual bee species (Table 7). These models, developed for the Dark European honey bee, serve as a reference for the evaluation of consistency of morphometric parameters of the lines covered by the conservation programs including the one for the Augustow M line.

According to Table 8, bees of the Augustow M line from the lead apiary are sufficiently consistent with the model, although values *z* are smaller than 2.1. However, the values are negative for the width of tergite 4 for all apiary types, which reveals the dwarfing trend. The analysis of mean values of the index of similarity, $\bar{y}$ (0 $<\bar{y}<3$), for three features—the width of tergite 4, the glossa length and the cubital index—shows that bees of the Augustow M line from all the three apiary types very closely resemble the reference population, the evidence of which is the value of the $\bar{y}$ ˂ 1 indicator.

Additionally, the mean values of morphological features of the Augustow M line bees were compared to the corresponding values provided by the reference for this line (Table 9). The mean values determined by the authors were within the reference range and the mean values of the cubital index and of the glossa length were 2% and 3% larger, respectively. Only the mean width of tergite 4 was 1.6% smaller than in the reference for the Augustow M line.

Based on the comparison of the mean values of morphological features of bees sampled from the lead, associate and conservation area apiaries, and for the overall population of the Augustow M line, to the morphological reference applicable to this line, the results were within the range of the reference. The mean cubital index value closest to the reference was found in bees from the conservation area apiaries; the difference was just 0.01. For the lead and associate apiaries, the value was larger by 0.04 and by 0.07, respectively. Bees from all the apiary types had the fourth tergite width smaller than the reference one, though the difference was smallest for bees from the associate apiaries (0.01 mm). The differences for the two remaining apiary types ranged from 0.018 mm to 0.05 mm. The mean values of the glossa length in bees from all the apiary types were smaller than the mean value of this parameter given in the reference and the differences were as follows: 0.182 mm for the lead apiary, 0.237 mm for the associate apiaries and 0.238 mm for the conservation area apiaries.

The authors compared the values of the coefficient of correlation between the bee size (defined by the sum of tergites 3 + 4) and the remaining parameters (wing length and width, cubital index value and glossa length) determined by the authors and by Bornus [53]. According to the authors, the coefficient of correlation between the wing width and the bee size is highly significant: *r* = 0.373 (*p* < 0.01). It is larger than that determined by Bornus [53]: r = 0.242 for F_0.01_.

Similarly, the coefficient of correlation *r* between the wing length and the bee size is different: *r* = 0.063 (F_0.05_), small but relevant, according to Bornus [53] vs. *r* = 0.276 (*p* < 0.01) determined by the authors. The correlation between the cubital index and the body size was very weak and negative, though highly significant, *r* = −0.030 (*p* < 0.01), according to the authors, while the value determined by Bornus [53] was higher, though also negative and highly significant, *r* = −0.230 (F_0.01_). Then, the values of correlation between the body size and the glossa length according to both the authors and Bornus [53] were very small: *r* = 0.191 (*p* < 0.01), significant, and *r* = 0.013 (F_0.05_), highly significant, respectively.

## 5. Conclusions

1. Bees of the Augustow M line have the features of the Dark European honey bee.
2. The values of the cubital index, glossa length and tergite 4 width of the Augustow M line are consistent with the morphological feature references valid for the *Apis m. mellifera*.
3. The program for the conservation of genetic resources of Dark European honey bees of the Augustow M line can be pursued based on the lead, associate and conservation area apiaries. The diversification of morphological features did not shrink during the study period.
4. Dwarfing trends were noted for the Dark European honey bee based on results of the authors’ research and on literature of the 1960s concerning *Apis m. mellifera* sizes in Poland measured by the width of abdominal tergite 4.

## Figures and Tables

**Table 1:** Description of morphometric features of Dark European honey bee of the Augustow M line.

Line	Feature	Number [n]	Range	Mean ± SE	±SD	Variation Coefficient [V]
Augustow	Wing length (mm)	6927	8.741–10.121	9.390 ± 0.002	0.172	1.83
	Wing width (mm)	6927	2.812–3.581	3.195 ± 0.001	0.103	3.24
	Wing form factor	6927	2.610–3.320	2.941 ± 0.001	0.083	2.8
	Cubital index (Goetze)	6927	1.029–2.678	1.664 ± 0.003	0.254	15.23
	Tergite 4 width (mm)	6927	1.885–2.821	2.284 ± 0.001	0.086	3.75
	Tergites 3 + 4 width (mm)	6927	4.012–5.365	4.764 ± 0.002	0.157	3.29
	Glossa length (mm)	6927	5.521–6.618	6.104 ± 0.002	0.157	2.57
Reference of morphological features *						
	Cubital index (Goetze)		1.25–2.00	1.63		
	Tergite 4 width (mm)		2.04–2.60	2.32		
	Glossa length (mm)		5.75–6.50	5.90		

* Reference of morphological features of the middle-European species valid for the Augustow M line in Poland [18].

**Table 2:** Description of morphological features of Dark European honey bee of the Augustow M line broken down by the lead, associate and conservation area apiaries in conformity with the organizational schemes of the conservation program.

Apiaries	Feature	Number [n]	Range	Mean ± SE	±SD	Variation Coefficient [V]
Lead	Wing length (mm)	4174	8.741–10.121	9.374 ± 0.003	0.165	1.76
	Wing width (mm)		2.812–3.528	3.171 ± 0.002	0.107	3.38
	Cubital index (Goetze)		1.029–2.678	1.665 ± 0.004	0.258	15.51
	Tergite 4 width (mm)		1.885–2.597	2.268 ± 0.001	0.092	4.05
	Tergites 3 + 4 width (mm)		4.012–5.263	4.741 ± 0.003	0.167	3.52
	Glossa length (mm)		5.521–6.618	6.082 ± 0.002	0.157	2.58
Associate	Wing length (mm)	1576	8.93–9.924	9.434 ± 0.004	0.162	1.72
	Wing width		2.962–3.581	3.244 ± 0.002	0.079	2.43
	Cubital index (Goetze)		1.062–2.659	1.695 ± 0.006	0.252	14.87
	Tergite 4 width (mm)		2.110–2.531	2.31 ± 0.002	0.06	2.59
	Tergites 3 + 4 width (mm)		4.258–5.197	4.808 ± 0.003	0.118	2.45
	Glossa length (mm)		5.673–6.588	6.137 ± 0.004	0.141	2.29
Conservation area	Wing length (mm)	1177	8.776–9.961	9.382 ± 0.006	0.199	2.13
	Wing width		2.959–3.474	3.214 ± 0.003	0.091	2.83
	Cubital index (Goetze)		1.098–2.604	1.620 ± 0.007	0.232	14.82
	Tergite 4 width (mm)		2.021–2.821	2.302 ± 0.002	0.079	3.45
	Tergites 3 + 4 width (mm)		4.299–5.365	4.786 ± 0.004	0.148	3.09
	Glossa length (mm)		5.609–6.611	6.138 ± 0.005	0.164	2.68
Reference of morphological features *						
	Cubital index (Goetze)		1.25–2.00	1.63		
	Tergite 4 width (mm)		2.04–2.60	2.32		
	Glossa length (mm)		5.75–6.50	5.90		

* Reference of morphological features of the middle-European species valid for the Augustow M line in Poland [18].

**Table 3:** Comparison of Dark European honey bee of the Augustow M line from the lead and associate apiaries.

Apiaries	Number [n]	Wing Length [mm]	Wing Width [mm]	Cubital Index (Goetze)	Tergite 4 Width [mm]	Tergites 3 + 4 Width [mm]	Glossa Length [mm]
		Mean	Mean	Mean	Mean	Mean	Mean
Lead	4174	9.376 ^b^	3.178 ^b^	1.663 ^a^	2.280 ^a^	4.7651 ^a^	6.100 ^a^
Associate	1576	9.405 ^a^	3.198 ^a^	1.641 ^a^	2.285 ^a^	4.774 ^a^	6.101 ^a^

^a, b^—Significant differences at *p* < 0.05.

**Table 4:** Comparison of the conservation area apiary to other apiaries for the Dark European honey bee of the Augustow M line kept based on the conservation area.

Apiaries	Number [n]	Wing Length [mm]	Wing Width [mm]	Cubital Index (Goetze)	Tergite 4 Width [mm]	Tergites 3 + 4 Width [mm]	Glossa Length [mm]
		Mean	Mean	Mean	Mean	Mean	Mean
Lead and associate	5750	9.382 ^b^	3.186 ^a^	1.657 ^a^	2.282 ^b^	4.768 ^a^	6.105 ^a^
Conservation area	1177	9.412 ^a^	3.174 ^a^	1.680 ^a^	2.295 ^a^	4.781 ^a^	6.097 ^a^

^a, b^—significant differences at *p* < 0.05.

**Table 5:** Relations between features of Dark European honey bee of the Augustow M line sampled from the lead and associate apiaries.

	Wing Length	Wing Width	Wing Form Factor	Cubital Index	Tergite 4 Width	Tergites 3 + 4 Width	Glossa Length
Wing length	1						
						
Wing width	0.509	1					
	<0.0001						
Wing form factor	0.026	−0.846	1				
	0.0447	<0.0001					
Cubital index	−0.006	−0.096	0.106	1			
	0.6398	<0.0001	<0.0001				
Tergite 4 width	0.276	0.373	−0.266	−0.030	1		
	<0.0001	<0.0001	<0.0001	0.0219			
Tergites 3 + 4 width	0.310	0.396	−0.270	−0.023	0.865	1	
	<0.0001	<0.0001	<0.0001	0.0802	<0.0001		
Glossa length	0.267	0.359	−0.251	−0.058	0.191	0.202	1
	<0.0001	<0.0001	<0.0001	<0.0001	<0.0001	<0.0001	

**Table 6:** Mean values of the cubital index obtained by various authors for the Dark European honey bee, including the Augustow M line, covered by the program for the conservation of genetic resources in Poland.

Author	Cubital Index	
	Acc. to Goetze	Acc. to Alpatov [%]
Gromisz & Bornus 1971 [54]	1.658 *	60.30
Gromisz 1972 [51]	1.626 *	61.50
Gromisz 1981 [55]	1.628 *	61.40
Gromisz & Platek 1999 [56]	1.600 *	62.50
Rostecki 2009 [36]—Augustow M line	1.850	54.80
The authors’ research—Augustow M line	1.664	60.10 **

*—Index values determined by the Alpatov method, converted into the Goetz method using Rostecki’s equations [36]. **—Index values determined by the Goetz method, converted into the Alpatov method using Rostecki’s equations [36].

**Table 7:** Mathematical-morphological models of bee populations for selected species in Poland [46].

Feature	Species		
	Middle-European	Crainian	Caucasian
Tergite 4 width	z = 24.2718x − 57.1845	z = 24.2718x − 55.8252	z = 24.2718x − 54.4175
Glossa length	z = 10.2042x − 62.3980	z = 10.2042x − 65.9184	z = 10.2042x − 71.3878
Cubital index	z = 0.311x − 19.082	z = 0.311x − 15.925	z = 0.311x − 16.983

z—Normalized value of the feature. x—Mean actual value of the feature of any hive.

**Table 8:** Values of morphological features covered by the reference for the Augustow M line, computed in conformity with the mathematical-morphological models for the population of the Dark European honey bee.

Line	Area	Feature			$\;\;\bar{y}$
		Tergite 4 Width [z]	Glossa Length [z]	Cubital Index [z]	
Augustow M	Lead apiary	−2.1361	−0.3531	0.0345	0.8412
	Associate apiaries	−1.1166	0.1978	−0.3343	0.5496
	Conservation area apiaries	−1.3108	0.2182	0.5014	0.6768

z$=\frac{x-\bar{x}}{S}$; y=|z|; $\bar{y}=\frac{1}{n}=|\frac{x_{1}-{\bar{x}}_{1}}{S}|$+…+$\left|\frac{x_{n}-{\bar{x}}_{n}}{S}\right|$; [54]. Z—normalization; S—standard deviation; $\bar{y}$—index of similarity of the honeybee colony to the model- range (0,3); N—number of features in the model; X1, Xn—features considered by the model.

**Table 9:** Comparison of consistency of the ranges and mean values of morphological features for the Augustow M line with the references valid for Poland.

Area	Feature/Consistency with the Reference											
	Cubital Index				Tergite 4				Glossa Length			
	Range	+/−	Mean	+/−	Range	+/−	Mean	+/−	Range	+/−	Mean	+/−
Lead apiary	1.03–2.68	−	1.665	+	1.89–2.60	−	2.268	+	5.52–6.62	−	6.082	+
Associate apiaries	1.06–2.66	−	1.695	+	2.11–2.53	+	2.310	+	5.67–6.59	−	6.137	+
Conservation area apiaries	1.10–2.60	−	1.620	+	2.02–2.82	−	2.302	+	5.61–6.61	−	6.138	+

+ Consistent, − non-consistent.

## Data Availability

All data are available from the corresponding author.
